# Bio-Inspired Microdevices that Mimic the Human Vasculature

**DOI:** 10.3390/mi8100299

**Published:** 2017-10-07

**Authors:** Md. Mydul Islam, Sean Beverung, Robert Steward

**Affiliations:** 1 Department of Mechanical and Aerospace Engineering, University of Central Florida, Orlando, FL 32816, USA; miku@Knights.ucf.edu (M.M.I.); K214beverungs@Knights.ucf.edu (S.B.); 2 Departments of Mechanical and Aerospace Engineering, College of Medicine, Burnett School of Biomedical Sciences, University of Central Florida, Orlando, FL 32816, USA

**Keywords:** endothelial cells, fluid shear stress, stretch, hydrogel, mechanical force, (polydimethylsiloxane) PDMS, blood vessels, blood brain barrier

## Abstract

Blood vessels may be found throughout the entire body and their importance to human life is undeniable. This is evident in the fact that a malfunctioning blood vessel can result in mild symptoms such as shortness of breath or chest pain to more severe symptoms such as a heart attack or stroke, to even death in the severest of cases. Furthermore, there are a host of pathologies that have been linked to the human vasculature. As a result many researchers have attempted to unlock the mysteries of the vasculature by performing studies that duplicate the physiological structural, chemical, and mechanical properties known to exist. While the ideal study would consist of utilizing living, blood vessels derived from human tissue, such studies are not always possible since intact human blood vessels are not readily accessible and there are immense technical difficulties associated with such studies. These limitations have opened the door for the development of microdevices modeled after the human vasculature as it is believed by many researchers in the field that such devices can one day replace tissue models. In this review we present an overview of microdevices developed to mimic various types of vasculature found throughout the human body. Although the human body contains a diverse array of vascular systems for this review we limit our discussion to the cardiovascular system and cerebrovascular system and discuss such systems that have been fabricated in both 2D and 3D configurations.

## 1. Introduction

The cardiovascular system is arguably one of the most functionally diverse systems in the body as it helps maintain homeostasis, by (1) regulating PH; (2) aiding in regulation of heat loss; and (3) aiding in immune response, therefore helping to protect the body against infection [1]. This geometrically complex system facilitates the transport of many important substances throughout the body and consists of blood vessels, the heart, and blood [1]. Blood consists of a mixture of water, cells, proteins, and other materials that are either dissolved or in suspension. The heart provides the majority of the working force required to propel blood through the body, while blood vessels provide the infrastructure and pathways for blood to travel throughout the body and acts as a semi permeable membrane selectively allowing the flow of material into and out of various tissues [1].

Blood vessels are composed of multiple biological layers that consist of either cells, basement membranes, or a combination of both. Blood vessel structure in general consists of the endothelium (inner layer), basement membrane, internal elastic lamina, smooth muscle (middle layer), external elastic lamina, and the tunica externa (outer layer). The endothelium is anchored to the basement membrane, which consists mainly of collagen fibers that provide blood vessel structure and strength while keeping them flexible. Veins and arteries have an additional layer of smooth muscle that controls blood vessel geometry [1] and an external sheath of elastic and collagen fibers called the tunica externa [1]. The tunica externa also can contain nerves and smaller blood vessels that support the tissues in larger blood vessel walls. However, capillaries only have a basement membrane and endothelium.

The discrepancies in blood vessel structure mentioned above are due to the physiological demands often imposed upon it on a routine basis. For example, the ascending aorta will be among the thickest vessels found in the body as they must be mechanically reinforced to bear the high pressures induced by blood propelled by the heart, while capillaries found in the lower extremities will be much thinner as they are further away from the heart and therefore bear lower pressures. In addition, beyond serving as a conduit for blood delivery, the thinner blood vessels are generally found in regions of the body where mass transport of essential nutrients and gasses across the vessel wall is important. The previously mentioned blood vessel functions are mentioned to highlight the fact that the vasculature is critical to the proper functioning of every major organ within the body. This fact has motivated many researchers to examine blood vessel structure, and function, but the physiological diversity of the human vasculature, cost in performing in vivo testing, and limited accessibility of human vascular tissue remains an issue in the field. To overcome this issue many researchers have begun to develop bio-inspired microdevices that mimic the human vasculature. Such devices have been developed to mimic a wide range of human vasculatures ranging from the arteries of the heart to the brain’s capillary system (called the blood brain barrier) in both 2D and 3D configurations. Therefore, we present here microdevices developed to mimic various types of human vasculature in 2D and 3D configurations.

## 2. 2D Microdevices

### 2.1. Probing Cellular Biochemical Response

As previously stated, the inner lumen of all blood vessels within the human vasculature is comprised of endothelial cells (ECs) only at the capillary level, but surrounded by additional layers of smooth muscle cells and fibroblasts at the arterial and venous level [2]. Furthermore, both blood cells and ECs experience a range of mechanical stimuli simultaneously, such as fluid shear stress, cyclic stretch, and hydrostatic pressure [3]. As a result, a host of microdevices has been developed in an attempt to elucidate the influence these mechanical stresses have on cell behavior as well as mimic the structural complexity of the vessels themselves. This has yielded many notable works that have demonstrated the influence of fluid shear stress on (endothelial cell) EC morphology, migration, proliferation, permeability, and gene expression [4,5]. Although vascular functions is now well understood to be influenced by both chemical and mechanical factors, a majority of the early microdevices developed focused on analyzing the chemical influence on cell behavior versus the mechanical influence. A review of such preliminary works is collectively presented by Fisher et al. [6] where EC response to physiological levels of fluid shear stresses (0.5–2 Pa) are proposed to be a result of one or more of the following mechanical sensors; integrins, G proteins, ion channels, intercellular junction proteins, receptor kinases, membrane lipids and the cytoskeleton [3,6]. While such experiments have increased our comprehension of EC mechanosensitivity a notable criticism is that they require high cell counts and high amounts of chemical reagents. Moreover, the efficacy of many of these systems are questioned due to their limitation of being able to investigate limited fluid shear stress regimes (steady, laminar flow) and their lack of ability to mimic the pulsatile nature by which most fluids flow through the vasculature [2,7].

**Figure 1:**
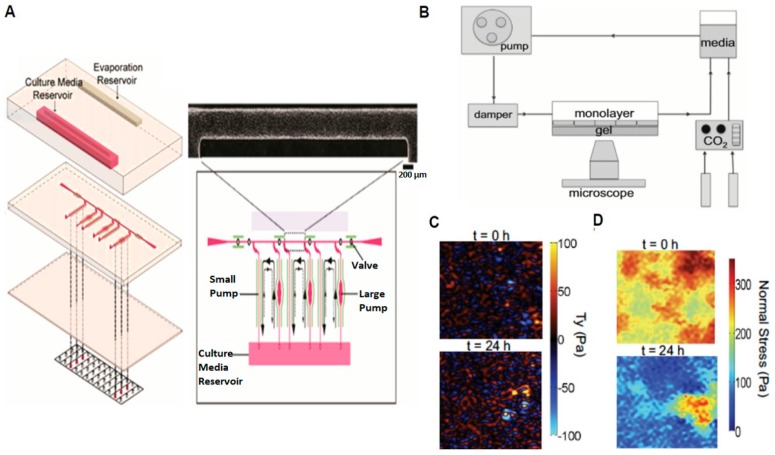
2D Microdevices Utilized to Study Fluid Flow, Intercellular Stress, and Contractile Forces on Endothelial Cells (ECs). (**A**) Schematic and close-up view of a braille pin device used to generate pulsatile flow on cells; (**B**) Schematic of experimental setup used to apply a steady, laminar fluid shear stress on ECs; (**C**) Contractile forces and (**D**) Intercellular stress generated by ECs after exposure to fluid shear stress (adapted from Song et al. [8] and Steward et al. [10]).

However, numerous microdevices have been developed to address these criticisms. Song et al. [8] presented a microdevice that utilized braille pins to generate various levels of pulsatile fluid shear stress simultaneously through micro-channels (Figure 1A). Their integrated, three layered microfluidic model is made from polydimethylsiloxane (PDMS) and has two pumps (small and large) capable of generating physiological levels of pulsatile shear stress ranging from 5 to 20 dyne/cm^2^ (Figure 1A). A 24-h time lapse comparison revealed EC orientation to not change significantly using the small pump (average shear stress <1 dyne/cm^2^, frequency 1 Hz), while EC orientation exhibited a decrease of orientation about 20 deg. using the large pump (average shear stress ~9 dyne/cm^2^, frequency 1 Hz). Chau et al. presented another microfluidic system with ten different non-pulsatile laminar shear stress levels (0.07–13 Pa) generated on a single chip [7]. ECs under both pulsatile [8] and non-pulsatile [7] flow exhibited elongation and alignment as well as an increase in expression of Von Willerband Factor (VWF). Rossi et al. [9] reported a tapered chamber design that provided pre-defined shear stresses ranging from 0.5 Pa to 1.5 Pa on ECs cultured in a single microchannel. Their experimental outcomes demonstrated that an increase in the shear-responsive translation factor KLF2 with the incremental increase of shear stresses along the channel [9].

### 2.2. Probing Cellular Biomechanical Response

While the above devices represent those used to derive biochemical outcomes on cell behavior, recent microdevices have been developed to better understand the influence mechanics has on cell behavior. In general, parallel plate flow chambers have been extensively used to quantify cellular contractile forces and intercellular stresses generated by a confluent monolayer. Shiu et al. reported a Rho-dependent increase in EC under laminar fluid flow [11] while other groups have reported an increase in cellular traction forces and intercellular stresses in a confluent EC monolayer [10]. Lam et al. [12] also presented a microdevice to mimic the vasculature and measure contractile forces generated by ECs under fluid flow. This device used flexible, micropost arrays to measure contractile forces and demonstrated fluid flow to increase EC contractility [13]. Our group has also developed a microdevice combined with traction force microscopy and monolayer stress microscopy to measure cell-generated contractile forces and intercellular stresses under a laminar fluid flow [10] (Figure 1B). Using our system we demonstrated laminar fluid flow to induce a dramatic decrease in intercellular stresses, yet negligible change in contractile forces (Figure 1C,D).

**Figure 2:**
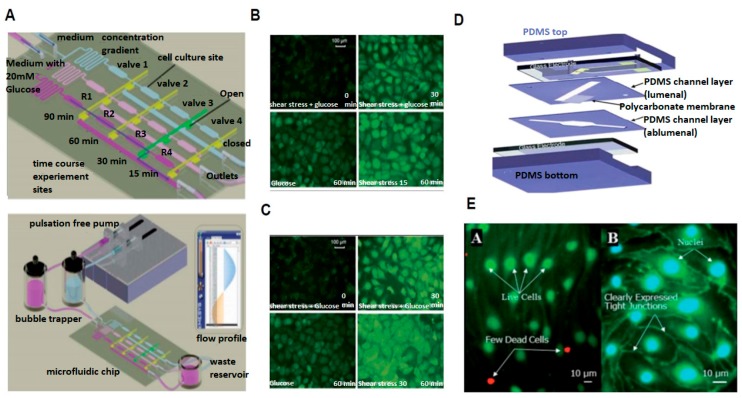
2D Microdevices Utilized to Study Glucose Exposure and the Blood Brain Barrier Under Fluid Flow. (**A**) Schematic of microfluidic chip for exposing cells to various glucose concentrations under fluid flow; (**B**) Fluorescent image of endothelial cell morphology and reactive oxygen species spatial distribution under low fluid shear stress; (**C**) Fluorescent image of endothelial cell morphology and reactive oxygen species spatial distribution under high fluid shear stress; (**D**) Schematic of μ-Blood Brain Barrier device showing; and (**E**) fluorescent images of cells cultured inside μ-Blood Brain Barrier device (adapted from Chin et al. [16] and Booth et al. [17]).

Mimicking flow in the human vasculature is difficult due to the complex shape of the vascular geometry, specifically in regions where flow is disturbed. Disturbed flow is a collection of broadly classified flow regimes that may consist of a low fluid shear stress, nonuniform flow direction, or shear stress gradient. In addition, disturbed flow has also been directly linked to many devastating diseases such as atherosclerosis, in-stent restenosis, aortic valve calcification, and thrombosis, for example [14]. Therefore, to gain insight into the influence disturbed flow has on cellular physiology and pathology appropriate microdevices have been developed. Estrada et al. [14] developed a microfluidic device capable of exposing ECs either non-reversing flow or reversing flow. Using this model, reversing flow conditions were observed to reduce both β-catenin expression and EC alignment when compared to non-reversing flow conditions [14]. Another microdevice developed by Sei et al. [15] was utilized to investigate endothelial response to various fluid flow conditions. In this study, human aortic endothelial cells (HAECs) were exposed to both laminar shear stress (LSS) and oscillatory shear stress (OSS) magnitudes of +10 dyne/cm^2^ and +10 and −9 dyne/cm^2^ at 1 Hz, respectively [15]. Fluorescent image intensities of the pro-inflammatory protein ICAM and adherens junction protein β-catenin were reported to be negligible under LSS, but were reported to have a frequency-dependent increase under OSS. In addition, endothelial monolayers were also reported to exhibit a frequency-dependent decrease in TEER under OSS when compared to LSS [15]. Another model by Chin et al. [16] investigated the impact of hyperglycemic conditions on ECs under various shear stress magnitudes (Figure 2A). This microdevice was fabricated using soft lithography and utilized two inlets to create a glucose concentration gradient in three separate micro-channels. Results revealed an increase in intracellular reactive oxygen species when high glucose conditions were coupled with fluid shear stress when compared to static conditions (Figure 2B,C) [16].

The pulsatile nature of blood flow induces cyclic strain and pulsatile flow upon the vessel wall. This has motivated the development of many microfluidic platforms aimed at simulating both previously mentioned mechanical regimes simultaneously. Zhou et al. [18] reported a two-layered microfluidic chip that consisted of multiple microfluidic channels with widths ranging from 20 μm to 500 μm covered by a thin, elastic, PDMS membrane. This thin membrane was used to apply a circumferential strain to cultured cells via hydrodynamic pulsation within the channel. This system created an artificial vessel and was reported to induce mesenchymal stem cell (MSC) alignment parallel to the microchannel and induce activation of the SMAD1/SMAD2 and Wnt/β-catenin pathways [18]. Another microfluidic system developed by Zheng et al. [19] also utilized a PDMS membrane to apply stretch and a peristaltic pump to simultaneously provide a laminar fluid shear stress in ECs and smooth muscle cells (SMCs) co-cultured on the membrane [19]. Utilizing this device, the combination of stretch and fluid shear stress induced increased cellular migration and adhesion compared to cells in the absence of mechanical force. The combination of stretch and fluid shear stress has also been implemented as a “dual mechanical force integration” device, developed by Steward et al. In this study a new vector logic gate notation was developed that allowed for the representation of cellular response to either fluid shear stress, stretch, or a combination of both forces via Boolean logic [20].

The microdevices mentioned above each have a common, underlying theme in that they have all primarily focused on understanding endothelial function, but the response of the blood cells that are carried through the vasculature are arguably equally as important. To this end, multiple microdevices have been developed to examine blood cell (erythrocytes, leukocytes, and platelets) responsiveness to mechanical forces as they flow through the vasculature [2]. A high throughput microdevice reported by Gutierrez et al. can deliver fluid shear stress levels ranging from 0.05 Pa to 5 Pa and was used to subsequently investigate the role of αIIbβ3 integrin on platelet adhesion [21]. Their microfluidic chip consisted of 8 parallel test chambers with one inlet and one outlet to deliver the fluid flow. The authors reported that platelets lacking αIIbβ3 integrin isolated from transgenic mice showed strong, impaired adhesion to the extracellular matrix at all fluid shear stress levels, but a point mutation in αIIbβ3 adhesion only reduced adhesion at intermediate and high shear stress levels [21]. Many microfluidic assays have also been developed to observe erythrocyte response under fluid shear stress. Lee et al. [22] developed a model to analyze and compare deformation index (DI) of red blood cells (RBCs) under extensional flow and conventional shear flow. Unlike shear flow, suspended deformable material in the liquid doesn’t rotate in the direction of extensional flow. Under simple shear flow RBC DI was about 0.55 at 20 Pa and in extensional flow DI was slightly higher at a much lower shear stress level of 6 Pa, proving efficacy of extensional flow in deforming RBC [22]. Yaginuma et al. [23] used a similar model and reached similar conclusions about RBC deformation under extensional flow. They also showed RBC deformability to be flow rate-dependent under extensional flow. Rodrigues et al. [24] used a cross-flow filtration device to separate RBCs and White Blood Cells (WBCs) and showed DI of RBCs to be about 0.44 ± 0.04 and WBCs DI to be about 0.1. Unlike previous models [22,23], this model has a recovery section where RBC DI reduced dramatically (0.24 ± 0.05) compared to WBC (0.06). These results suggest that WBCs are less deformable compared to RBC when subjected to similar extensional flow [24]. Wan et al. [25] used a specially designed microchannel to observe erythrocyte response in narrow vessels. Their channel had a uniform height (42 μm) and uniform width (width = 100 μm) with a constriction in the middle (width < 100 μm). ATP release from erythrocytes was observed to occur when fluid shear stress duration exceeded 6 ms [25]. A similar system was also used by Forsyth et al. to demonstrate that cytoplasm viscosity and lipid membrane composition are responsible for erythrocyte deformation under fluid shear stress [26]. Not only erythrocytes, but leucocytes were also shown to be responsive to mechanical stimulus. A study by Yap et al. [27] forced neutrophils through a narrow microchannel made from PDMS and observed neutrophil deformation and elongation while migrating through the channel. The authors designed their microdevice to mimic the pulmonary capillaries and reported applying mechanical stress to neutrophils to cause an increase pseudopodal activity and reduce neutrophil shear modulus [27].

### 2.3. Probing Cellular Barrier Response

In addition to the cardiovascular system inspired microdevices discussed thus far, another important class of microdevices that has recently gained a considerable amount of attention are those modeled after the cerebrovascular system. An essential component of the cerebrovascular system is the blood brain barrier (BBB), which is a highly specialized vascular system that protects the brain from invading pathogens and helps regulate the central nervous system (CNS). With, a thorough understanding of the BBB is essential to gaining insights into various BBB-related diseases such as Alzheimer’s and Parkinson’s, for example. This intimate relationship between the BBB and neurodegenerative disease has motivated the development of many BBB microdevices. One such notable device is presented by Booth et al. [17]. This “μBBB“ device contains a porous polycarbonate membrane sandwiched at the center of the device with an integrated trans-endothelial electrical resistance (TEER) measuring system to measure endothelial barrier resistance (Figure 2D). In addition, there are two perpendicular crossing channels to provide laminar fluid flow. Both asctrocyte and EC cell lines were co-cultured in this system. Using this model, the authors reported ZO-1 junction expression in ECs as evidence that ECs cultured in this system in fact express typical bbb expressed in vivo (Figure 2E). To validate these results TEER values were found to be comparable to in vivo measurements as well, reflecting this device’s potential to mimic BBB physiological vascular behavior [17]. Griep et al. [28] reported an additional BBB microdevice that stimulated blood brain barrier endothelial cells both biochemically and mechanically using tumor necrosis factor-alpha (TNF-α) and fluid shear stress. Their model was reported to maintain EC culture for up to 7 days and express conventional BBB markers for up to day 4 in culture. Another model developed by Yeon et al. [29] allowed permeability assays to be performed to evaluate drug delivery to the CNS via the BBB. Their model trapped ECs in patterned holes and supplied astrocyte-conditioned media (ACM) at a constant flow rate of 10 μL/h to achieve fluid shear stresses of 0.28 to 8.19 dynes/cm^2^. After incubating for 2 h with ACM, ECs displayed decreased permeability.

## 3. 3D Microdevices

The 2D microdevices such as the ones mentioned above are predominantly used as they are often much simpler to design and build relative to 3D microdevices. However, 3D microdevices can provide a more structurally relevant representation of the complex geometries known to exist within the body [30,31,32,33]. In addition, cellular properties and behaviors such as cell stiffness, alignment, viability, migration, proliferation, and even differentiation have all been demonstrated to be different in 3D relative to 2D [31,34,35,36,37] As a result, 3D bio-inspired microdevices has become an emergent field.

Earlier microsystems developed mainly consisted of using either micro-milling or injection-mold based techniques to fabricate a 3D matrix, which is generally composed of a hydrogel, extracellular matrix, or combination of both [32,33,37,38,39,40]. An added advantage of the gel-based systems is their stiffness can be mechanically tuned to any tissue of interest meaning the gels could be fabricated such that they are as soft as fat or as hard as bone, for example. These 3D systems can also allow for the investigation of 3D cellular processes such as angiogenesis [37,38,40,41].

**Figure 3:**
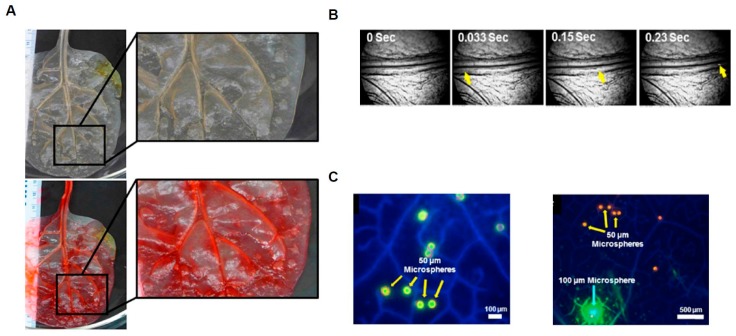
The 3D Microdevice fabrication Using Spinach Leaf Scaffolds. (**A**) Optical Image of spinach leaf before and after perfusion; (**B**) Video frames of microspheres traveling through scaffolding; (**C**) Fluorescence images of microspheres traveling through scaffolding (adapted from Gershlak et al. [34]).

Beyond the commonly used mico-milling or mold-derived systems mentioned above, additional microdevices have been developed using novel techniques that utilize biological intermediates as a design template. An elegant example of this is present by Gershlak et al. In this study decellularized spinach leaves were injected with fibronectin, which was subsequently injected with endothelial cells and smooth muscle cells for revascularization (Figure 3) [34]. After 24 h of incubation cell growth within the interior of the revascularized vessel was confirmed with the use of a fluorescent tracer (Figure 3).

**Figure 4:**
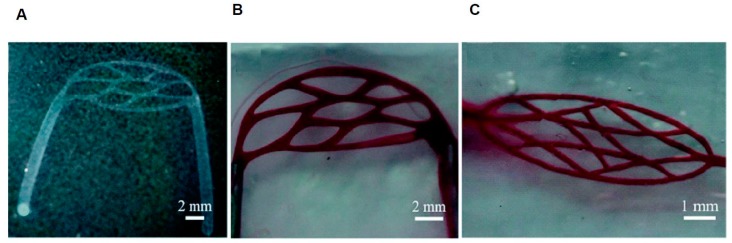
The 3D Microcirculation Network. (**A**) Image of microcirculation network template; (**B**,**C**) Image of microcirculation network perfused with dye (adapted from Wang et al. [32]).

**Figure 5:**
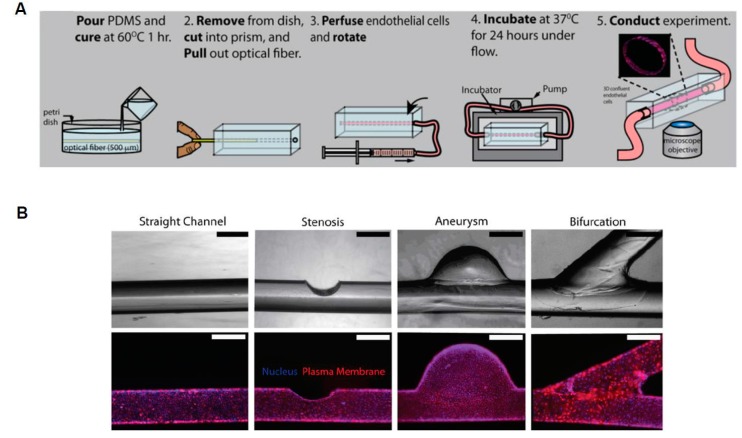
Microfabrication of 3D vasculature using optical fiber. (**A**) Schematic of microfabrication process; (**B**) Phase contrast and fluorescent images of normal and abnormal vascular channels (adapted from Mannino et al. [33]).

Complex, 3D models can also be constructed to replicate the multiple, intersecting blood vessels that occur throughout the body. In this regard, 3D printing can assist in this process by creating inserts that hydrogel molds can be polymerized around. These inserts can be designed to be potentially any desired shape and generally consist of a sacrificial layer that can be dissolved away after the hydrogel polymerizes. The utilization of 3D-printed molds allows for the studying of diseased vessel models due to the considerable flexibility provided by this method (Figure 4) [32]. Furthermore, 3D microdevices have the add advantage of allowing for the fabrication of structural pathologies not possible in 2D. A study presented by Mannino et al [33]. utilized an optical fiber to develop a vascular system that could simulate either an aneurysm or stenosis (Figure 5) [33]. This fiber was placed in a hydrogel and removed once the gel cured. The fabricated aneurysm created a region of low stress were the vessel ballooned out, while the narrowing of the vessel emulating stenosis caused an increase in shear stress at the walls in the area where the vessel narrows. These areas of low and high shear stress were observed to correlate with the expression of cell adhesion molecule expression [33].

The 3D microdevices have also been developed to study cancer cell metastasis since blood vessels also aid in the transport of cancer cells from one tissue to another [1]. Wong et al. presented such a device that consisted of a cancer cell and endothelial cell co-culture system with the goal of providing information regarding cancer cell migration speed through tissue and across the endothelial layer into vascular circulation. [42]. This system consisted of 3 cylindrical collagen gels (150 μm diameter) in parallel lined with endothelial cells that were maintained at a physiological level of fluid shear stress between 12 and 15 dyne/cm^2^. The collagen gel was subsequently seeded with cancer cells to visualize cancer metastasis. Breast cancer cells were observed to migrate through the 3D vasculature matrix towards the artificial lumen at a maximum speed of 1.1 μm/min and an average speed 0.3 μm/min, but cancer cell migration slowed rapidly as they reached the endothelium. Furthermore, using this system cancer cell migration was proposed to depend on nutrient availability [42].

## 4. 2D vs. 3D Microdevices: Advantages and Disadvantages

The 2D and 3D microdevices we present here illustrate the many configurations and methods that can be utilized to fabricate microdevices. In this section we present advantages and disadvantages of the microdevice fabrication methods discussed in this paper (Table 1) and highlight important advantages and disadvantages of using 2D and 3D microdevice models in general (Table 2). A major advantage of 2D models is their compatibility with many commonly used lab-on-a-chip technologies. Utilization of lab-on-a-chip technologies allows for fast experimental data acquisition, real-time experimental observation, and quick chip fabrication at low costs [2,37,38]. Such characteristics have made this among the most desirable methods of choice for in vitro disease and drug delivery studies. Common fabrication methods include photolithography and soft lithography. Soft lithography consists of fabricating structures using elastomeric materials and molds [43], while photolithography consists of using light to transfer patterns to light-sensitive compounds coated on stiff, thin substrates. Both soft lithography and photolithography allows for the development of complex geometrical shapes with variable cross-sections. Furthermore, computational modeling of 2D models is generally less computationally expensive and more consistent experimental image data is more readily acquired since only one plane needs to be imaged [44,45,46].

Although 2D microdevices are low cost and allow for fast data acquisition, they do not fully replicate the complex 3D blood vessel geometry. 3D microdevices may therefore provide more accurate analysis of how cells behave under various mechanical and chemical stimuli [30,35]. With 3D microdevices complex vascular networks can be fabricated and simulated [30,33,37,38,40]. The need to mimic complex vascular geometries within the body have yielded many novel microfabrication methods. The majority of these methods general consist of fabricating a mold that replicates vascular dimensions found in vivo [32,33,42]. One such mold replication method consists of using a wire, cord, or glass rod to create a microvessel template cast out of an elastomeric material. Small deformations can be introduced to this template to create non-uniform vascular geometries that can mimic an aneurysm or stenosis, for example [33,42]. Photolithography and soft lithography has also been used to create 3D microdevices, but their fabrication is generally more time consuming when compared to 2D microdevice fabrication. This is because in general separate device layers must be independently fabricated and subsequently bonded together to form a complete structure. The lithography methods mentioned above have also been combined with micromilling methods to improve efficiency and help provide more realistic vascular geometries [37,47].

## 5. Future Directions

The desire to mimic the human vasculature’s complex geometry and associated vascular pathologies has been motivated by many researchers’ goal to find cures and novel therapies for some of the most devastating diseases known to mankind. In this review, we have discussed a plethora of microdevices such as PDMS-based devices with polycarbonate membrane for blood brain barrier models, hyperbolic chambers to induce extensional flow on red blood cells, and ecm-based microdevices for studying cancer metastasis, for example. While each of these microdevices have to some extent successfully mimicked the in vivo microenvironment and contributed significantly to the field there are future directions we believe will yield even greater advancements. Such future directions include developing microdevices capable or mimicking the multiple stimuli cells are known to experience in vivo. Such stimuli include chemical, mechanical, thermal, and electrical. While, the mode and nature of stimuli the cell will experience will depend on its physiological location, the cell will most likely experience at least two stimuli at any given point in time. Neurons for example utilize electrical and chemical signals for neurotransmission. Furthermore, cells may also experience multiple modes of a single stimuli as endothelial cells experience a simultaneous fluid shear stress and stretch with each heartbeat.

## 6. Conclusions

This review focused on current 2D and 3D microdevices of the human vasculature and provided key outcomes utilized from these devices. In addition, we explored current materials and methods utilized to build such devices as well as conventional cell culture protocols and microfabrication techniques. In addition, to the review provided here we point the reader’s attention to notable additional reviews on vascular microdevices that specifically expand into the fields of vascular tissue engineering [44], mechanobiology [2], vascularization strategy [45], and vasculature-on-a-chip [46]. Although these reviews go beyond the scope of what we present here we highlight these topics as we believe the future of bioinspired microdevices to consist of systems capable of probing the complex biochemical and biomechanical cellular response.

## Figures and Tables

**Table 1:** Fabrication techniques advantages and disadvantages.

Fabrication Method	Advantages	Disadvantages
Lithography	Can create complex vascular networks [32].	Traditionally has squared geometries.
Micro Milling	Can be used with lithography [37].	Resolution depended on milling machine [37].
Angiogenesis	Cellular action created channels [38].	Difficulty creating consistent geometry [38].
Rod/Wire Template.	Can Create simple anatomically abnormal vessels [33,42].	Unable to create complex networks [42].

**Table 2:** 2D vs. 3D model advantages and disadvantages.

Model Dimensions	Advantages	Disadvantages
2D	Lab on chip technology [2,33,38]. Fast data processing times and simulations.	Lacks 3D geometric cell considerations [30].
3D	Able to more accurately model 3D cell interactions [30]. Able to model anatomical abnormalities [33].	May require stacked images for analyses. More complex data analysis [33,40].
